# Visualisation of Sentinel Lymph Node with Indium-Based near Infrared Emitting Quantum Dots in a Murine Metastatic Breast Cancer Model

**DOI:** 10.1371/journal.pone.0044433

**Published:** 2012-08-30

**Authors:** Marion Helle, Elsa Cassette, Lina Bezdetnaya, Thomas Pons, Agnès Leroux, François Plénat, François Guillemin, Benoît Dubertret, Frédéric Marchal

**Affiliations:** 1 Université de Lorraine, Centre de Recherche en Automatique de Nancy (CRAN), UMR 7039, Vandoeuvre-lès-Nancy, France; 2 CNRS, Centre de Recherche en Automatique de Nancy (CRAN), UMR 7039, Vandoeuvre-lès-Nancy, France; 3 Centre Alexis Vautrin, Research Unit, Vandoeuvre-lès-Nancy, France; 4 Laboratoire de Physique et d’Etude des Matériaux, Ecole Supérieure de Physique et de Chimie Industrielles, CNRS, Université Pierre et Marie Curie, UMR 8213, Paris, France; 5 EA4421 Signalisation, Génomique et Recherche Translationnelle en Oncologie (SiGReTO), Université de Lorraine, Vandoeuvre-lès-Nancy, France; National Institute of Health, United States of America

## Abstract

Due to its non-invasiveness, high temporal resolution and lower cost, fluorescence imaging is an interesting alternative to the current method (blue dye and radiocolloid) of sentinel lymph node (SLN) mapping in breast cancer. Near-infrared (NIR) emitting cadmium-based Quantum Dots (QDs) could be used for this purpose; however, their wide application is limited because of the toxicity of heavy metals composing the core. Our recent work demonstrated that indium-based QDs exhibit a weak acute local toxicity *in vivo* compared to their cadmium-based counterparts. In the present study we confirmed the weak toxicity of CuInS_2_/ZnS QDs in different *in vitro* models. Further *in vivo* studies in healthy mice showed that In-based QDs could be visualised in SLN in a few minutes after administration with a progressive increase in fluorescence until 8 h. The quantity of indium was assessed in selected organs and tissues by inductively coupled plasma – mass spectroscopy (ICP-MS) as a function of post-injection time. QD levels decrease rapidly at the injection point in the first hours after administration with a parallel increase in the lymph nodes and to a lesser extent in the liver and spleen. In addition, we observed that 3.5% of the injected indium dose was excreted in faeces in the first 4 days, with only trace quantities in the urine. Metastatic spread to the lymph nodes may hamper its visualisation. Therefore, we further performed non-invasive fluorescence measurement of QDs in SLN in tumour-bearing mice. Metastatic status was assessed by immunohistology and molecular techniques and revealed the utmost metastatic invasion of 36% of SLN. Fluorescence signal was the same irrespective of SLN status. Thus, near-infrared emitting cadmium-free QDs could be an excellent SLN tracer.

## Introduction

The metastatic status of the sentinel lymph node (SLN) is a determinant predictor of recurrence and survival [1] and directs treatment modality [2]. The current method for localising the SLN consists of an injection of a blue dye and a radiolabelled colloid [3]. However, both procedures present some disadvantages, such as the use of radioactivity and allergic reactions [4]. The ideal time for injection of the radiocolloid is also not well established, thus requiring interdepartmental scheduling for this two-step procedure. New methods based on near-infrared (NIR) fluorescence imaging are now under consideration. NIR imaging offers multiple advantages such as deep tissue penetration and high temporal resolution. An additional benefit is related to reduced hospitalisation, since the SLN could be detected just few minutes after NIR tracer injection.

Indocyanine green (ICG) is the first NIR emitting tracer that has been used for the detection of the SLN. ICG coupled to human serum albumine (HSA), thus reaching a hydrodynamic diameter of 7 nm, has been successfully tested for the detection of SLN in an animal model of spontaneous melanoma [5]. Likewise, clinical studies have already proven the good detection rate of SLN with this technique in various tumour types [6]–[11]. Nevertheless some problems related to ICG-HSA size and fluorescence stability upon irradiation persist. Fast photobleaching has been observed and the small size of ICG-HSA results in a rapid migration from SLN to adjacent nodes a few minutes after injection [7]. Recently, the cyanine dyes, including ICG have been embedded into lipid nanoparticles (Lipidots) and demonstrated a fast NIR fluorescence-based detection of SLN in healthy mice [12]. However, the biodistribution and *in vivo* toxicity related to cyanine dyes release was not assessed [12]. Quantum dots (QD) could also be considered as a valuable alternative marker with regard to their high fluorescence quantum yield, resistance to photobleaching and adaptable size [13], [14]. Several extensive reviews address the remarkable photochemical and photophysical properties of QDs [14]–[16].

The first QD studies have shown the possibility of SLN mapping through the skin after an intradermal or subcutaneous injection of cadmium-based QDs in healthy mice or pigs [17], [18], spontaneous melanoma in mini-swine [5] or xenografted mice [19]. However, the metastatic status of SLN has never been addressed in these tumour-bearing animals [5], [19]. The visualisation of the SLN in a metastatic model of cancer is of key importance, since a distal obstruction of lymphatic channels and nodes by tumour metastases could prevent the migration of tracers to SLN, affecting its identification [20].

A major concern with the *in vivo* applications of QDs is their toxicity, since the surrounding inorganic shell is degraded *in vivo*, releasing heavy metal ions and as such inducing oxidative stress and cell death [21], [22]. The storage of QDs in the reticulo-endothelial system and absence of renal elimination for QDs >5.5 nm are also problematic [23], [24]. Toxicity could be significantly reduced using cadmium-free QDs. Metals, like copper and sulphur in combination with indium, could be more attractive for NIR fluorescence-guided SLN imaging. As we have recently shown, CuInS_2_/ZnS QDs possess all requisites for SLN NIR-imaging, given their emission wavelength in the NIR window (650–800 nm), their hydrodynamic diameter (∼20 nm) and significantly reduced acute local toxicity compared to CdTeSe/CdZnS QDs [25].

Therefore, we undertook further investigations of CuInS_2_/ZnS QDs biodistribution with inductively coupled plasma – mass spectroscopy (ICP-MS) up to 7 days after subcutaneous injection in healthy mice. Selected organs were followed until 3 months after injection. We further evaluated the QDs-based NIR SLN visualisation *in vivo* in a metastatic model of orthotopic syngenic murine breast cancer.

## Materials and Methods

### Ethics Statement

The animals received care in accordance with established guidelines of the Federation of European Laboratory Animal Science Associations. Animal procedures were performed in compliance with national guidelines and with approval of regional ethics committee in animal experiment “Nancy-Lorraine – Nord-Est”. All surgery was performed under ketamine/xylazine or isoflurane gaseous anesthesia, and all efforts were made to minimise suffering.

### Quantum Dots Characteristics

The synthesis and physico-chemical characteristics of Cd- and In-based QDs were reported earlier [17], [25]. Briefly, batches of QDs were composed of CdTeSe/CdZnS or CuInS_2_/ZnS core/shell with a maximum fluorescence emission at 680 nm and 780 nm respectively. QDs were then encapsulated in 33% PEG2000-COOH/66% PEG2000 lipid micelles and purified by ultracentrifugation.

### Cell Culture

4T1 cell line (ATCC, Manassas, VA, USA) derived from a Balb/c spontaneous mammary carcinoma was cultivated in RPMI medium containing 9% inactivated foetal calf serum, 1% penicillin (10,000 UI)-streptomycin (10,000 µg/mL) and 2 mM glutamine. MRC-5 cell line (ATCC, Manassas, VA, USA) derived from a normal lung tissue was cultivated in Minimum Essential Medium (MEM) containing 9% inactivated foetal calf serum, 1% penicillin-streptomycin, 2 mM glutamine and 1 mM sodium pyruvate. All culture flasks were kept in a humidified incubator at 37°C and 5% CO_2_.

### Cell Toxicity Tests

#### Viability assay

Effects of QDs on the viability of MRC-5 cells were evaluated using the MTT assay [21]. Briefly, MRC-5 cells were plated into 96-well plates and after 24 h the culture medium was replaced by 200 µL QDs suspension at concentrations of 0–200 nM. After the end of incubation (24 h or 48 h), cells were washed, 50 µL MTT (2 mg/ml) was added to each well and incubated at 37°C for 3 h. The water-insoluble formazan crystals were solubilised with dimethyl sulfoxide (DMSO) and optical density was read on a microplate photometer (Multiskan Ascent, Thermo Labsystems, Finland) at 540 nm. The results were expressed as the concentration of QDs inducing 50% of cell death (IC_50_) compared to the untreated controls.

#### Haemolysis test

The method used was reported elsewhere [26]. Briefly, mice blood stabilised with EDTA was obtained by intracardiac puncture before sacrifice. Red blood cells were suspended in phosphate buffered saline (PBS) (negative control), deionised water (positive control) or different QDs dilutions in PBS, ranging from 0 to 200 nM. The solutions were incubated for 2 h at 37°C, before centrifugation. The absorbance spectra of the supernatants at 541 nm were measured with a spectrophotometer (Lambda 35, Perkin-Elmer, Courtaboeuf, France). The percentage of haemolysis of each sample was calculated by subtracting the absorption of the negative control from the absorption of the sample, divided by the difference in absorption between positive and negative controls.

### Animals

Ten-week-old female Balb/cOlaAnN mice (Harlan, Gannat, France) were kept in a 12 h light/dark cycle and had access to food and water *ad libitum*. A specific purified diet (TD.94045, Harlan Teklad, Madison, WI, USA) was used to reduce tissue autofluorescence in the NIR region. The model of lymphatic metastasis in mice grafted with 4T1 mouse mammary carcinoma cells was reported earlier [27], [28]. Under anaesthesia, mice (n = 53) were injected subcutaneously in the first right fat pad with 40 µL of 10^6^ 4T1 cells in water solution supplemented with 5% glucose. Mice were sacrificed when the tumour reached the ethical volume of 1,000 mm^3^ (about 21 days) and the tumour and the axillary lymph nodes (ALNs) were removed. Mice were divided into two groups: in the first group (n = 25), tumours and right axillary lymph nodes (RALNs) were fixed in acetic formalin alcohol (AFA) for immunohistochemical (IHC) analysis and in the other group (n = 28), tumours and RALNs were rapidly frozen at –80°C for molecular biology analysis.

### Biodistribution Study

Seven groups of animals (n = 3 per group) were used for biodistribution study: one control group received PBS and was sacrificed 7 days after injection and six experimental groups, where animals were injected with 20 pmol CuInS_2_/ZnS QDs and sacrificed at 1 h, 4 h, 8 h, 72 h, 7 days and 3 months after injection. Mice were injected subcutaneously (s.c.) in the distal part of the right anterior paw with 20 µL of PBS or 20 µL of a 1 µM-QD solution. The right paw was kneaded to improve product migration and the RALN were imaged *in vivo* 5 minutes after injection. After sacrifice, organs (ALNs, lateral thoracic lymph nodes (LTLNs), brain, bladder, intestine, spleen, pancreas, stomach, kidneys, liver, heart, lungs and injection point) were removed, weighed and stored at –20°C prior to analysis. Blood samples were collected through cardiac puncture in heparinised tubes before sacrifice.

### Near-infrared Fluorescence Imaging

*In vivo* optical imaging of QDs was performed using a Fluobeam™700 NIR imaging system (Fluoptics, Grenoble, France) described previously [17]. This system is composed of a 690 nm-emitting laser and a highly sensitive charge-coupled device camera with a 750-nm long-pass emission filter. Semi-quantitative data can be obtained from the fluorescence images using the ImageJ 1.44 software. A segmentation protocol (Max Entropy method) was applied to threshold the image for each organ. Fluorescent signals were expressed in arbitrary units (A.U.) as the mean intensity of pixels of the threshold organ.

### Inductively Coupled Plasma – Mass Spectroscopy (ICP-MS)

A Varian 820 MS instrument (Varian, Les Ulis, France) was used to perform ICP-MS analyses [17]. Briefly, all samples (organs, blood and excretions) were dissolved with 70% HNO_3_ and heated to 90°C until total mineralisation. Each mineralised sample was solubilised in 25 mL milli-q water and analysed by ICP-MS. The limit of indium quantification was 0.05 µg/L. Five samples of 1 mL QD solutions at 20 nM (20 pmol) were analysed to correlate for indium concentration.

### Immunohistochemistry Staining of Lymph Node Section

Immunohistochemistry was carried out on 5 µm-thick AFA-fixed paraffin-embedded sections. After rehydratation and antigen retrieval a primary rabbit monoclonal antibody against mouse cytokeratin 19 (CK19, Epitomics, Burlingame, CA, USA) diluted at 1∶200 was applied. After overnight incubation at 4°C, the endogenous peroxidases were inactivated and a polymer detection system anti-rabbit Histofine (Nichirei Biosciences, Tokyo, Japan) was applied for 30 min. Peroxydase activity was revealed with the Novared kit (Vector laboratories, Burlingame, CA, USA) in accordance with the supplier’s instructions. Sections were finally counterstained with Mayer’s Hematoxylin (Dako, Trappes, France).

### RT-qPCR

RNAs from lymph nodes were purified using an AllPrep DNA/RNA MiniKit (Qiagen, Courtaboeuf, France) in accordance with the supplier’s instructions. cDNA was synthesised using an iScript™ Reverse Transcription kit (BioRad, Marnes-la-Coquette, France) in accordance with the manufacturer’s recommendations. A real-time qPCR was performed in iCycler iQ™ (Biorad, Marnes-la-Coquette, France). The reaction mixture contained 12.5 µL of iQ™ SYBR® Green Supermix, 1.5 µL of 5 µM forward primer, 1.5 µL of 5 µM reverse primer, 4.5 µL of DNase RNase free water and 5 µL of cDNA. The PCR program was 95°C for 5 min, 50 cycles of 95°C for 10 s, 65°C for 30 s, 95°C for 10 s and 60°C for 30 s with a final extension at 60°C for 5 min. The list of the primers is summarised in Table 1 [29], [30]. The housekeeping gene ß-actin was used as an internal control for the quantity of cDNA and results were expressed as the ratio CK19/ß-actin. The maximum ratio CK19/ß-actin (6.43×10^−3^) of ALN from healthy mice (n = 21) was used as a threshold: SLN with CK19/ß-actin ratio below this value were considered as non-metastatic, whereas those with greater ratios were considered to be metastatic.

**Table 1:** Sequence of primers used for real-time qPCR of cytokeratin 19 and â actin.

Primer		5¢ → 3¢	Reference
CK19	Forward	TGATCGTCTCGCCTCCTACT	[29]
	Reverse	CAAGGCGTGTTCTGTCTCAA	
ß-actin	Forward	CGTGGGCCGCCCTAGGCACCA	[30]
	Reverse	TTGGCCTTAGGGTTCAGGGGGG	

### Statistical Analysis

Means and standard errors (SD) were determined using standard software, whereas statistical significance between groups (Mann-Whitney test) was calculated with StatView software (SAS Institute Inc, Cary, NC, USA); *p* values <.05 were considered statistically significant.

## Results

### Toxicity Tests

Normal MRC-5 human fibroblasts were exposed to increasing concentrations of CdTeSe/CdZnS- or CuInS_2_/ZnS-based QDs during 24 h or 48 h. MTT-assessed cytotoxicity depended on the core element and time of contact with cells (Table 2). For Cd-based QDs, the IC_50_ values vary from 21 nM to 8 nM depending on incubation time, whereas the same toxicity was achieved for In-based QD at concentrations of 108 nM and 76 nM (Table 2). IC_50_ values were thus approximately five times lower with CdTeSe/CdZnS QDs than for In-based QDs.

**Table 2:** Cytotoxicity of CdTeSe/ZnS or CuInS_2_/ZnS QDs on normal fibroblasts.

IC_50_ (nM)	24 h	48 h
QD CdTeSe/CdZnS	21.40±4.42	8.41±3.29
QD CuInS_2_/ZnS	108.29±23.66	75.68±6.28

MRC-5 cells viability was assessed by MTT (3-(4,5-dimethylthiazol-2-yl)-2,5-diphenyltetrazolium bromide) assay.

Concentration of QDs causing 50% of cell death (IC_50_) was measured 24 h or 48 h after exposure.

Data are mean ± SD (n = 6 per condition).

**Figure 1:**
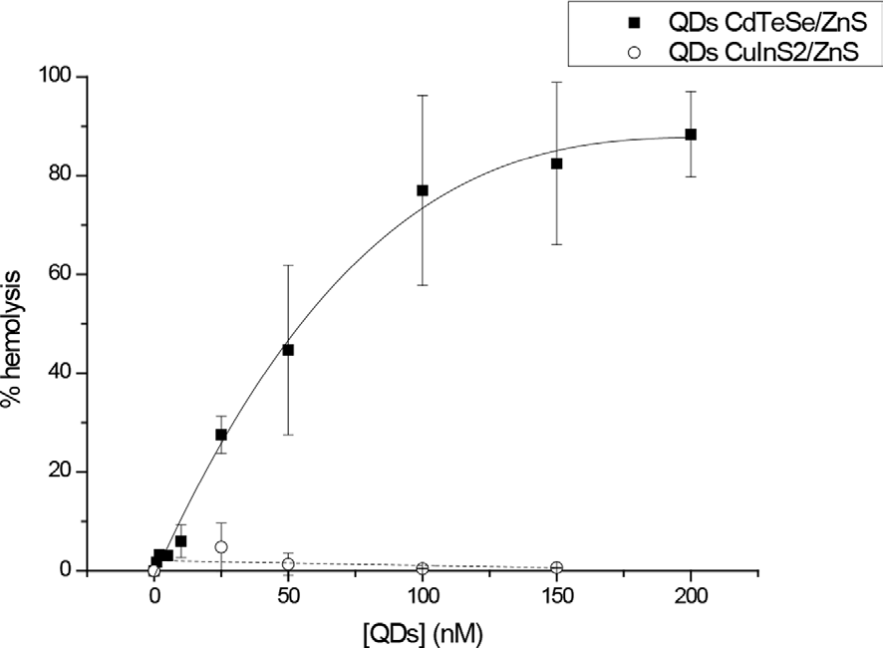
Haemolysis of red blood cells after a 2 h-exposure to CdTeSe/ZnS or CuInS_2_/ZnS QDs. Red blood cells were incubated for 2 h at 37°C in buffer containing increasing concentrations of CdTeSe/ZnS (close square) or CuInS_2_/ZnS QDs (open circle) and the absorbance of the supernatant was measured at 541 nm. Percentage of haemolysis is expressed as the amount of haemoglobin release induced by QDs compared to that induced by deionised water. Data are mean ± SD (n = 3).

We further measured QDs-induced haemolysis of red blood cells (RBC). RBC were incubated for 2 h with increasing concentrations of QDs and the haemoglobin release was assessed by measuring the absorbance of the supernatant (Fig. 1). Already 25 nM of Cd-based QDs induced 28% haemolysis with a rapid increase in haemolytic activity up to 75% at 100 nM and further stabilisation. No haemolysis was detected for In-based QDs, irrespective of concentration up to 150 nM (Fig. 1).

### Detection of the Regional Lymph Node

**Figure 2:**
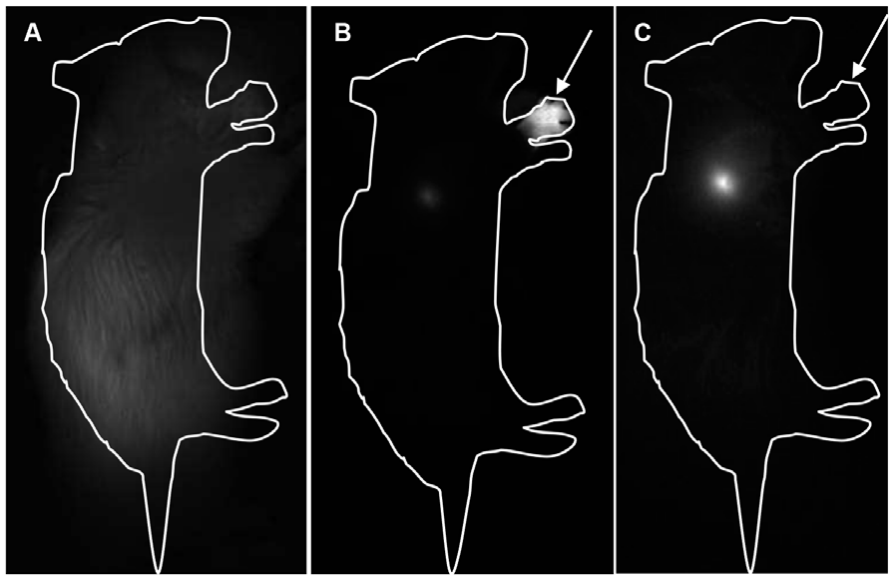
*In vivo* non-invasive fluorescence imaging of mice before or after QDs injection. Mice were subcutaneously injected with 20 pmol of CuInS_2_/ZnS QDs in the right anterior paw and imaging was performed before (a) or 5 minutes after (b, c) administration. b, injection point (arrow) was not hidden; c, injection point was hidden for a better RALN visualisation. Near-infrared imaging was performed with Fluobeam™ system (exc. 690 nm, em. 700–850 nm). Exposure times were 100 ms (a), 5 ms (b) and 10 ms (c).

**Figure 3:**
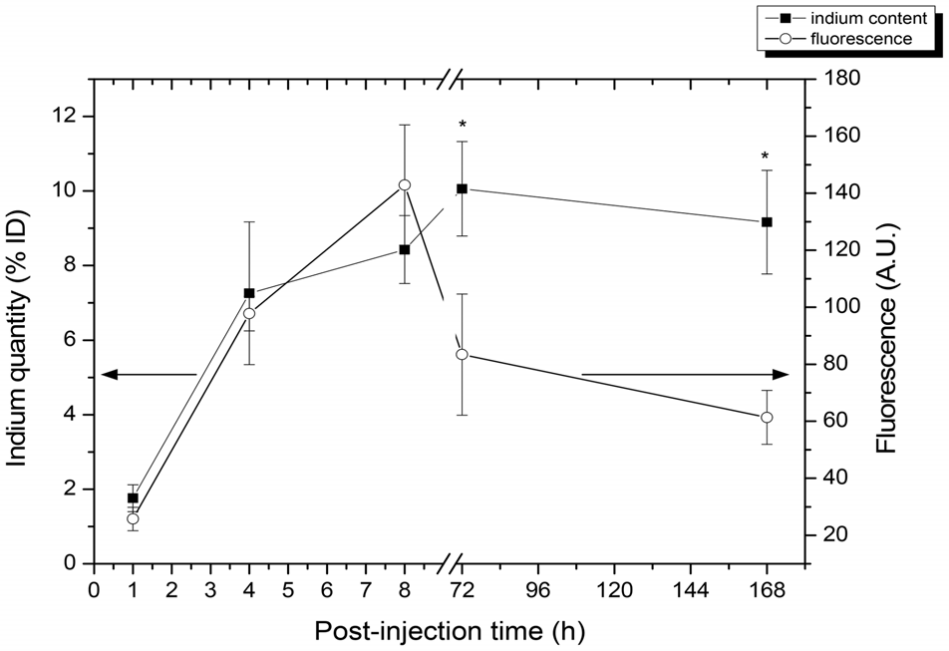
Assessment of QDs fluorescence intensity and indium content at different post-administration times. Kinetics of QDs fluorescence (open circle) and In content (close square) in the RALN after s.c. administration of 20 pmol of CuInS_2_/ZnS QDs in mice. The indium quantity was measured by inductively coupled plasma – mass spectroscopy (ICP-MS) and expressed in percentage of injected dose (% ID). Data are mean ± SD (n = 3 per group) and indium quantity values marked with an asterisk were significantly different from corresponding fluorescence values (*p*<.05).

After s.c. injection of 20 pmol of CuInS_2_/ZnS QDs in the paw, RALN could be visualised already after 5 min (Fig. 2b, c). For better visualisation, the injection point of QDs was hidden, thus enabling a precise localisation of RALN (Fig. 2b). Figure 3 displays the intensity of QDs fluorescence in RALN *in vivo* during seven days after injection along with the quantification of indium content in the same mice at the same time intervals. Fluorescence signals and In content in RALN demonstrate similar increase (r = 0.88) from 1 to 8 hours post-injection, followed by a rapid drop of fluorescence intensity from 8 to 72 h and a slow decrease until the end of observation (168 h) (Fig. 3). The In content however remains stable (∼9% injected dose-ID) from 8 h until 168 h (Fig. 3).

### Biodistribution and Excretion

**Figure 4:**
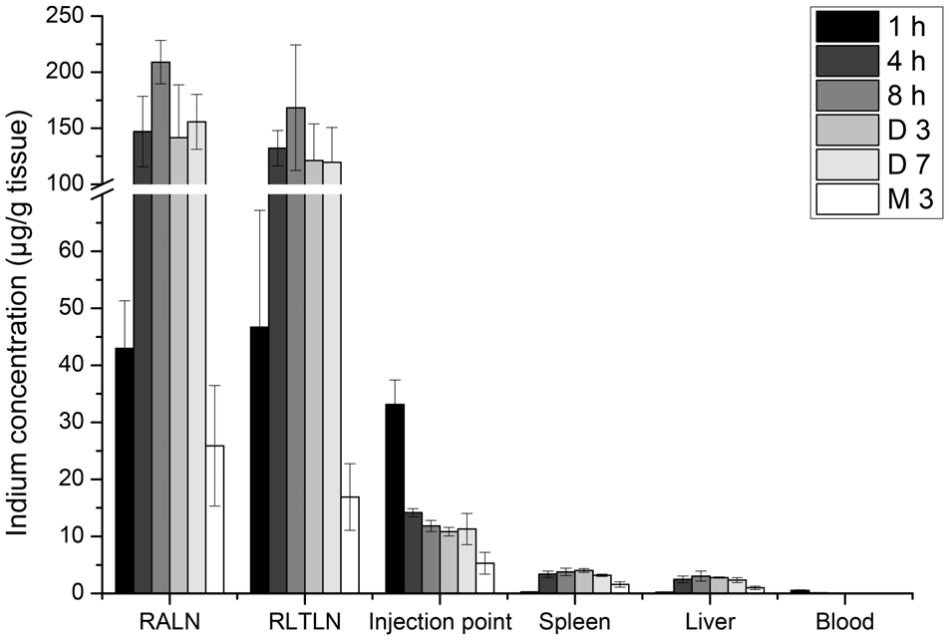
Biodistribution of QDs in selected organs and tissues in function of time after injection. Mice were subcutaneously injected with 20 pmol of CuInS_2_/ZnS QDs in the right anterior paw. Blood samples were collected through cardiac puncture. Mice were then sacrificed at 1 h, 4 h, 8 h, 3 days, 7 days and 3 months after injection and organs were subjected to mass spectroscopy (ICP-MS). The indium concentrations in RALN, RLTLN, injection point, spleen, liver and blood were expressed as the quantity of indium (in µg) per gram of tissue or per milliliter of blood. Data are mean ± SD (n = 3 per group).

**Figure 5:**
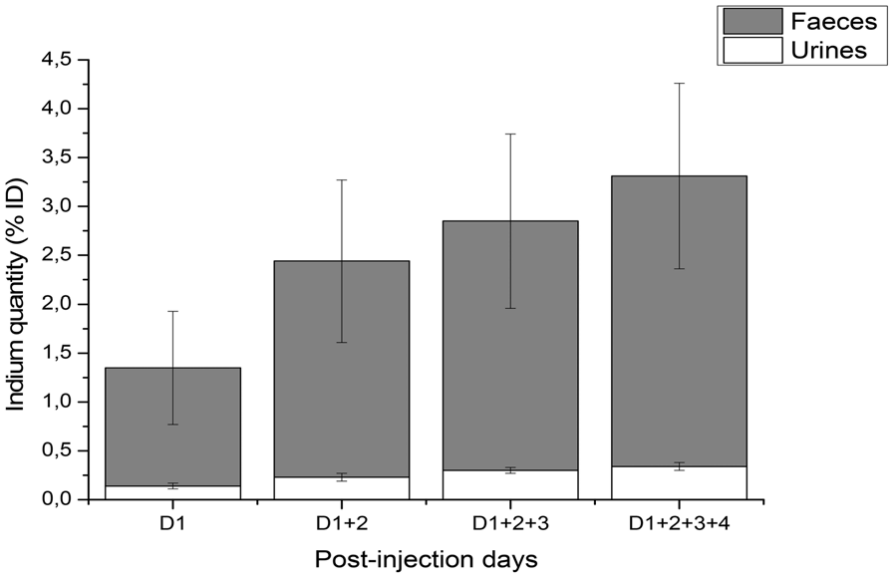
Cumulative indium content in excretions of mice after subcutaneous QDs administration. Mice were subcutaneously injected with 20 pmol of CuInS_2_/ZnS QDs, urine (white) and faeces (gray) were collected daily and subjected to mass spectroscopy (ICP-MS). The indium contents were cumulated every day and were expressed as percentage of injected dose (% ID). Data are mean ± SD (n = 3 per group).

As shown in Figure 4, the In concentration is similar in right lymph nodes (RLNs) and injection point 1 h after injection (∼40 µg In/g tissue) whereas no indium could be detected in liver and spleen. At 4 h after injection, the In concentration decreases at the injection point (14.16±0.71 µg In/g tissue), with a simultaneous considerable increase in the RLNs (147.08±31.42 µg In/g tissue and 132.18±15.77 µg In/g tissue for RALN and RLTLN respectively) and a slight increase of In concentration in both liver (2.46±0.60 µg In/g tissue) and spleen (3.36±0.56 µg In/g tissue). No variations were found for indicated organs until 7 days; however, a 2 times decrease of In was observed 3 months after injection. We also demonstrated the presence of indium in the plasma samples (0.54±0.10 µg In/g tissue) 1 h post-injection with only traces quantities of In at all other observation times. The clearance of QDs was assessed in the measurement of the cumulative doses of In in urine and faeces during the first 4 days after injection. Negligible quantities of indium (0.34±0.04% ID) were detected in urine, whereas In excretion with faeces reached 2.97±0.95% ID during the first 4 days (Fig. 5).

### Mapping of SLN in Metastatic Model with In-based QDs

Fifty-three mice bearing 4T1 tumours were subjected to RALN *in vivo* fluorescence measurements before their sacrifice. CK19 detection in extracted lymph nodes was performed by immunohistochemical analysis (n = 25) and by RT-qPCR (n = 28).

**Figure 6:**
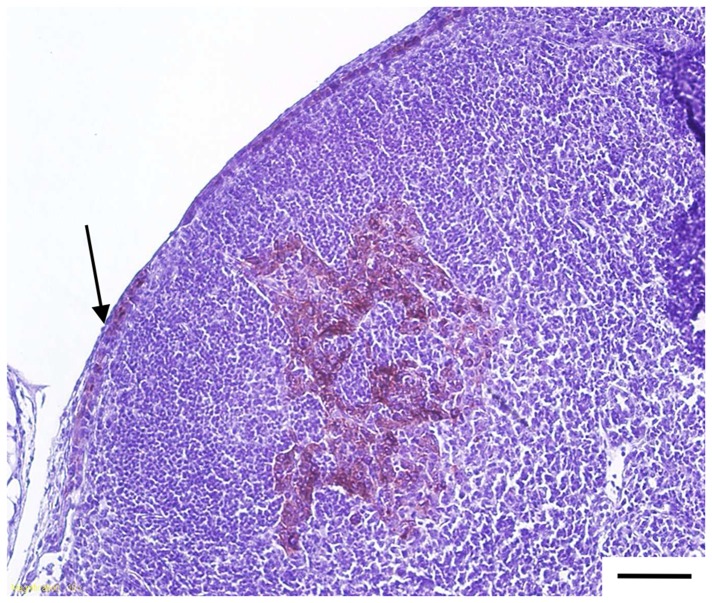
Histology of sentinel lymph node sections of tumour-bearing mice. Sections were subjected to CK19 immunohistochemistry and hematoxylin staining. Black scale corresponds to 100 µm, the arrow indicates the metastatic invasion of the cortical sinus.

Of 25 lymph nodes analysed by immunohistochemistry, 20 were metastasis free, whereas isolated tumour cells and metastatic nodules were detected in respectively three and two mice. Figure 6 displays an example of immunohistological section of metastatic lymph node. The metastasis detection by RT-qPCR performed in 28 lymph nodes demonstrated the presence of metastasis in 36% of SLN (Table 3).

**Table 3:** Mapping of sentinel lymph node (SLN) of tumour-bearing mice 15 min after sc. injection of 20 pmol of CuInS_2_/ZnS QDs in the right anterior paw.

Techniques of CK19 detection	Nodal status	Node affected	*In vivo* SLN fluorescence (A.U.)
IHC	+	20.00% (5/25)	21.90±7.20
	−	80.00% (20/25)	15.73±4.19
RT-qPCR	+	35.71% (10/28)	19.17±7.28
	−	64.29% (18/28)	19.90±9.45

Metastatic cells were detected in lymph node by CK19 expression using immunohistochemistry (IHC, n = 25) or molecular biology analysis (RT-qPCR, n = 28).

Data are mean ± SD.

All lymph nodes, which revealed CK19 expression, were pooled and referred as positive SLN (Table 3). The fluorescence intensity from each SLN was correlated to its metastatic status. As follows from Table 3, no statistical difference in fluorescence intensity was detected between positive and negative SLN (*p* = 0.1733). The mean fluorescence intensity in non-metastatic lymph nodes was averaged to 17.71±7.38 (n = 38), whereas positive SLN yielded 20.15±7.10 (n = 15) (Table 3).

## Discussion

NIR imaging could be an interesting alternative to the current method using blue dye and radioactive colloid for the visualisation of SLN in breast cancer surgery. Despite the excellent *in vivo* fluorescence properties of Cd-containing QDs [15], their toxicity, due to the release of Cd^2+^ ions in the biological environment is a serious drawback for clinical applications [31]. We have recently considered indium-based QDs, which are characterised by a bright fluorescence and a reduced acute local toxicity in lymph node [25]. Indeed, the onset of inflammation occurred at a 10 times higher concentration for CuInS_2_/ZnS QDs than for their Cd-containing counterparts [25].

In the present paper we extended the comparative study of toxicity between two types of QDs. Haemolytic capacity is regarded as a reference test to study nanoparticles interaction with blood [32]–[34]. Whereas CdTeSe/CdZnS QDs induce 50% haemolysis at a concentration of 56 nM, no haemolysis was found at all applied concentrations of CuInS_2_/ZnS QDs (Fig. 1). Mitochondrial activity of fibroblasts subjected to QDs demonstrated 5 to 10 times reduced toxicity of CuInS_2_/ZnS compared to cadmium-based QDs (Table 2). These results are similar to the cytotoxicity of fibroblasts subjected to cyanine (Cy5)-loaded Lipidots (IC_50_>75 nM) [12]. They are also in agreement with previous studies which suggest that indium-based QDs are less toxic than their cadmium-based counterparts and are thus more suitable for biomedical applications [35], [36]. A weak *in vivo* and *in vitro* toxicity of In-based QDs encouraged us to further test these nanoparticles in healthy and tumour-bearing mice.

QD-induced fluorescence in the RALN could be detected as early as 5 min after s.c. injection of 20 pmol of CuInS_2_/ZnS QDs in the paw (Fig. 2). The presence of QDs in the RALN has been confirmed by the measurement of indium content by ICP-MS (Fig. 3). An increase in both parameters was noted in the first hours post-injection indicating the integrity of the QDs. While the In content, as observed by mass spectrometry, remained stable with time, we observed a decrease in fluorescence intensity starting at 8 h post-injection. This could be related to the bio-degradation of QDs in an oxidative environment, despite capping of the QD cores by ZnS. It is possible that the ZnS capping loses a sulphur atom, leaving the core vulnerable to oxidation with the formation of oxides and eventually loss of QD fluorescence [21].

Potential sites of QD toxicity and elimination can be deduced from our biodistribution study. After s.c. injection in the paw, QDs strongly accumulate in the RLN (Fig. 4). QD retention is confined to regional lymph nodes only since no fluorescence was visible in other lymph nodes (cervical, inguinal, etc) pointing out that QDs do not migrate further into the lymphatic system. This observation could be advantageous in clinical settings since one of the pitfalls of SLN biopsy consists of the false positive samples related to the fast migration of the dye across the lymphatic chain. A fast increase in In concentration in lymph nodes from 1 h to 4 h after injection is consistent with the decrease of In concentration at the injection point in the same time span. Despite the fact that a major part of In was retained in the lymph nodes, no signs of toxicity could be detected in theses organs [25]; also the mice did not present any signs of abnormal behaviour or weight loss for up to 7 days after injection (data not shown). Similarly, Castronovo *et al.* observed that intravenous injection of hydrated indium comparable concentrations (0.4 mg In/kg body weight) yielded only a diffuse and mild inflammation in the liver, without noticeable damage to the kidneys [37].

In the clinical context, the resection of the injection site, which is the original source of QDs migration, can be performed within the first 15 min following injection, thus minimising QD accumulation in non-lymphatic sites.

The cumulative dose of In was 10 times higher in faeces (∼3% ID) compared to urine (∼0.3% ID) (Fig. 5). Faecal excretion of In after intratracheal or oral instillation of InP in rats was demonstrated earlier [38] and was suggested as a major route of elimination. The size of our nanoparticles is also in favour of hepatobiliary excretion. For renal excretion, the nanoparticles must have a hydrodynamic diameter smaller than 5.5 nm in order to be filtered by the kidney [24], whereas that of CuInS_2_/ZnS QDs used in our study reaches ∼20 nm [25]. Conflicting data were reported on heavy metal elimination in pre-clinical models. A high recovery of In was observed in faeces (73% ID) of rats after intratracheal administration of InP [38], while CdSe/ZnS QDs were sequestered in the liver of rats (36.4%ID) after i.v. injection and were not detected in the excretions during several weeks [39]. At three months after In-based QD injection, the quantity of indium in the liver was reduced by more than 50% (3.0±0.9 vs. 1.0±0.3 µg In/g tissue, Fig. 4), thus further supporting hepatobiliary elimination. However, in order to translate these findings to humans, long-term experiments are required in pre-clinical models.

It seems interesting to make an approximation of the remaining quantity of QDs in clinical setting. A good visualisation of the SLN with Cd-based QDs was reported after an injection of 400 pmol in 35 kg of Yorkshire pigs [18]. Considering that SLN were located at the depth of 1 cm in pigs, whereas the depth of SLN localisation in humans is of 3–6 cm, the dose of 2.4 nmol CuInS_2_/ZnS QDs could be required for SLN visualisation, i.e. 900 µg indium. A large part of QDs will be eliminated during the excision of both SLN and tumour burden at 1 h post-administration. Our present study demonstrated that the remaining quantity of In in the body of mice at 1 h after injection is about 17% (*ca.* 157 µg). The daily In consumption in humans is about 8 µg/day/person according to the National Toxicology Program [40]. Thus, compared to the annual In consumption (2920 µg), the remaining quantity of In in the body after QDs injection represents 5%. Except of occupational exposure to inhaled indium [41], [42], toxicology studies of indium in human body are sparse. Indium and indium oxide are not listed as human carcinogens by different official organisations (US Occupational Safety and Health Administration, Canadian Centre for Occupational health and safety, European Economic Community, etc). It may be therefore supposed, that such a small increase in In concentration in the body is not harmful. Further, given the extremely low rate of photobleaching of QDs, the fluence rate of the imaging system may be increased at least one order, resulting in a significantly reduced QDs injected dose and a decreased remaining In concentration.

Metastatic invasion of lymph nodes may modify the uptake of tracers [20], [43], [44]. Therefore, the possibility of fluorescence mapping of lymph nodes upon their distal obstruction by tumour metastases is of major importance. The metastatic status of lymph nodes was assessed by CK19 detection using immunohistochemistry and molecular biology approaches. CK19 is a member of the intermediate filaments found in the intracytoplasmic cytoskeleton of epithelial tissues [45] and is considered as a good marker for metastatic involvement of lymph nodes [46]. The qRT-PCT technique, which is more sensitive than immunohistochemistry, showed 35.7% of positive nodes *vs.* 20.0% detected by IHC (Table 3). The observed level of metastatic invasion is similar to that reported in the clinical study, conducted in 274 breast cancer patients where 36.4% positive and 63.5% negative lymph nodes were detected [47]. Fluorescence measurements demonstrated that all positive nodes could be visualised with NIR emitting QDs and no statistical difference in fluorescence intensity was established between positive and negative nodes (Table 3). Thus, the migration of QDs into the lymphatic system is not affected by the presence of metastatic cells, even when they are in the cortical sinus (Fig. 6).

In summary, indium-based QDs presenting a reduced *in vitro* and *in vivo* toxicity, are good tracers for SLN NIR fluorescence mapping in a metastatic murine breast cancer model. Further improvements of surface chemistry are nevertheless required to achieve faster body elimination of CuInS_2_/ZnS QDs.
